# The Anatomy of the Nerves of the Uterus

**Published:** 1841-04

**Authors:** 


Art. XII.

*The Anatomy of the Nerves of the Uterus.* By Robert Lee, m.d. f.r.s., Physician to the British Lying-in Hospital, &c. *With Two Plates.* —*London*, 1841. Folio, pp. 8.

This work is divided into two chapters, the first of which is devoted to a sketch of the history of the subject, and the second to a detail of the author's dissections of the nerves of the uterus. Reflecting on the remarkable functions of the uterus, its nervous system might *a priori* be expected to be much developed; yet, hitherto, little more than a few nervous filaments have been demonstrated in it. We must therefore consider that a worthy labour in which Dr. Lee has engaged, and for the communication of the results of which he is entitled to our best thanks. From the sensibility and contractile power of the uterus, Galen inferred that it must be supplied with nerves, but he says they are extremely small, compared with the size of the organ. The description which Vesalius gives of the nerves of the uterus, is very imperfect. It appears that Willis traced the hypogastric and sacral nerves to the neck of the uterus, but no farther, and that he was unacquainted with the spermatic nerves. In De Graaf's seventh plate, the trunk of a nerve is represented passing on each side into the posterior surface of the neck of the unimpregnated uterus, and ramifying like the branches of a tree over the body and fundus. Saltzman and Reuss, Vater, Rast, Maier, Daventer, and Winslow, all state that the uterus has nerves, but their works contain no new facts on the subject. J. G. Walter's first plate gives a very defective view of the nerves of the uterus in the unimpregnated state ; a few small filaments only being seen passing into the lower part of the orifice and cervix from the upper part of the hypogastric plexus. The fundus and body of the uterus are left covered with peritoneum, and the spermatic nerves are not traced. Haller appears likewise to have confined his attention to the nerves of the unimpregnated uterus, and with little more success than Walter and other anatomists before him had done.

Dr. William Hunter was the first anatomist who examined the nerves of the gravid uterus, and who suspected that they enlarge during utero-gestation, in some proportion like the vessels. Mr. John Hunter, however, denies that the nerves of the uterus are increased in the smallest degree during pregnancy ; but neither he nor his brother have left any preparations to demonstrate the accuracy of their respective statements. From his assumption that the nerves of the uterus are not enlarged in the time of pregnancy, Mr. Hunter infers that the nerves and brain have nothing to do with the actions of a part.

Dr. Lee is not aware that, except those now to be described, any preparations exist, or ever have existed in this country, showing the nerves of the uterus dissected, either in the unimpregnated or gravid state. Some anatomists believe that it is impossible for the nerves of a part to increase under any circumstances ; an assertion confuted in the clearest manner by the dissections by Mr. Newport of the nerves of insects. The same authors think that the intense pain and violent periodical contractions during labour do not depend upon the nerves, but upon some incomprehensible *vis insita* in the organ.

Professor Tiedemann has published a description of the nerves of the uterus, with two engravings. In the first plate, the spermatic nerves are seen on both sides, accompanying the spermatic arteries to the ovaria. The spermatic veins have not been represented, and it is not stated whether any nerves accompany these veins to the ovaria. The uterine arteries are seen running along the posterior surface of the uterus, and anastomosing near the ovaria with the spermatic arteries. The uterine nerves proceeding from the hypogastric plexuses, are seen accompanying the uterine artery, and spreading out on the posterior surface of the uterus, like the branches of a tree, nearly in the same manner as represented by De Graaf. The uterine veins have been removed, and all the nerves which accompanied them. The fundus and body of the uterus, down to a line drawn across it, a little lower than the ovaria, are covered with peritoneum. In the second plate, the same parts of the uterus are invested with peritoneum; some small branches proceeding from the superior part of the left hypogastric plexus, are seen accompanying the uterine artery, and spreading in an arborescent form on the side of the uterus. The uterine veins and nerves which covered them are also not represented. Professor Tiedemann believes that the nerves of the uterus enlarge after puberty, and become slender in old age, and that they increase both in number and magnitude during pregnancy, as Dr. Hunter had suspected. In the works of Osiander, Lobstein, Boivin and Duges, Cruveilhier, Velpeaù, &c. nothing new has been added to our knowledge of the subject.

Such was the state of our knowledge of the nerves of the uterus when Dr. Lee undertook his researches. The following abstract of the dissection of a gravid uterus in the sixth months will give a general idea of the distribution of the nerves of the uterus as ascertained by him:

Each of the hypogastric plexuses, after giving off several branches to the ureter, rectum, and uterus, descends to the side of the neck of the uterus and terminates in a large oblong ganglion. This ganglion receives communicating branches from the sacral nerves, chiefly the third. From the inner surface of the ganglion, numerous small white nerves are given off to the neck of the uterus, some of which ramify under the peritoneum, and others pass deep into the muscular coat. From the anterior and inferior borders of the ganglion, many large nerves are given off to the bladder and vagina, and from its posterior margin to the rectum. The nerves which come off from the hypogastric plexus before the termination of the latter in the ganglion at the cervix uteri, form a plexus around the ureter and uterine vessels, and prolongations of this plexus pass both to the anterior and posterior surface of the body of the uterus, and are then continued into peculiar membrani form plexuses, with which numerous branches from the ganglia at the neck of the uterus, and from the spermatic plexuses, may be distinctly observed to be continuous. In regard to these plexuses, Dr. Lee remarks:

"From the form, colour, and general appearance of these plexuses on the body of the uterus, and the resemblance they bear to ganglionic plexuses of nerves, and from their branches actually anastomosing and coalescing with the hypogastric and spermatic nerves, I was induced to conclude, on first discovering them, that they were nervous plexuses and constituted the special nervous system of the uterus." (p. 6.)

The following is Dr. Lee's account of the nerves of the gravid uterus in the fourth month, as recorded in his fifth dissection.

"In October, 1840, I finished the dissection of a gravid uterus of four months, all the arteries and veins on the right side of which are completely filled with red and blue injection; and the whole nervous system of the uterus more perfectly displayed than in any of the preparations already described. The uterus was removed from the body of a woman who died in St. George's Hospital, from an external injury, and the fœtus and its appendages were expelled a few hours before death. The nerves were traced while the uterus was covered with rectified spirit.* An artery of considerable size filled with injection is seen accompanying the hypogastric nerve, and passing along with its branches through the hypogastric plexus to the ganglion at the cervix. In this course, the artery is seen ramifying upon the trunk of the hypogastric nerve, and the most minute branches of the hypogastric plexus. The sacral nerves passing into the ganglion are also accompanied with an artery, which is likewise injected, and which passes through the centre of the ganglion. These nerves are a little smaller than in the uteri of nine months. The ganglion is thick, large, and distinct, of an oblong form, about three quarters of an inch in diameter, and consisting of gray and white matter. From its inferior border, three large bundles or masses of nervous fibres are sent off, which present an appearance resembling the pes anserinus of the portio dura. The posterior of these subdivides into numerous small branches, accompanied with arteries which supply the rectum and back part of the vagina. The middle of these great nerves proceeding from the ganglion, likewise accompanied with arteries, ramifies upon the side of the vagina, and the anterior upon the bladder, around the entrance of the ureter. From the hypogastric plexus, before it enters the ganglion, and from the inner surface of the ganglion, numerous large and small branches of nerves are given off to the neck of the uterus, some of which accompany the blood-vessels towards the fundus, and others spread out under the peritoneum. All these are likewise accompanied by injected arteries. From the inner border of the ganglion, a broad nervous band is sent off, which passes on the outside of the ureter, and another on the inside, which unite and completely surround the ureter. From these united nervous bands, many large branches are sent to the back part of the bladder, and into the anterior part of the cervix uteri. The course of these branches can be easily traced by their injected arteries. On the lower and anterior part of the cervix uteri, over the mesial line, there is a thick membranous expansion, into which these nerves enter from both hypogastric plexuses and ganglia. From the sides and upper part of this membrane, there are given off innumerable filaments, apparently nervous, which unite on the sides of the uterus, with the nerves accompanying the blood-vessels, and with the spermatic nerves, and some of which pass out with the round ligaments. These are likewise accompanied with minute arteries, as all the nerves are on the right side of the uterus, entering the ganglion and passing out from it. The nerves and ganglion on the left side correspond in appearance with those of the right, and the greater number of the spermatic nerves on both sides accompany the spermatic veins."† (p. 7.)

* It is only under spirit that dissections of this kind can be properly made. So long a time is necessarily occupied, that if water were used, the parts would putrefy before the dissection could be completed. To attempt tracing the nerves of such a structure as the uterus except under a clear liquid, would be vain.—Rev.

† "Analogy led me to suspect," says Dr. Lee, (p. 7,) "that many branches of nerves would also be found, on examination, to accompany the veins of the spermatic cord, and to ramify upon their coats. Mr. James Dunn, at St. George's Hospital, undertook, at my request, to ascertain if this were the fact, and in July last he made three preparations, in which the nerves are seen covering the veins as they pass out of the testicle. It appears from these, that a much greater number of nerves accompany the veins, than the artery in the spermatic cord. The nerves in these preparations, form a great plexus around the veins, and are traced into the testicle."

Whether Dr. Lee is correct in considering as nerves all the bands and filaments he has met with in his dissections, and which we believe are faithfully delineated in the plates, it is not competent for us to say, nor is it for any one who has not carefully gone through the investigation of the whole subject himself; a task we are aware of no little pains and labour. In these remarks, we allude particularly to the membraniform plexus under the peritoneum, on the body of the uterus, into which there is an evident continuity of branches from what are indisputably nerves. This structure is evidently the same as that alluded to by Lobstein, in the following quotation which Dr. Lee gives from his monograph on the sympathetic nerve: " Hac occasione," says Lobstein, " monendum esse duco, quod, tunica uteri externa sublata, multæ fibræ occurrunt, quæ vario modo sese decussant, et, ope telæ cellulosæ laxæ, tam inter se, quam substantiæ uterinæ profundiori atque densiori, uniuntur. Hæ fibræ, quorum indolem ignoro, facile pro continuatis nervorum ramulis habentur, a quibus vero, non solum ratione directionis atque crassitiei majoris, sed etiam ratione figuræ suæ magis complanatæ, differunt."

It is evidently also the same structure which is described by Madame Boivin and Professor Duges as " a filamentous tissue between the peritoneum and muscular coat of the uterus, and fibres extending longitudinally 'en avant et en arrière sur la region médiane du viscère de son corps aux moins,' " (see p. 4,) but which, as Dr. Lee remarks, they have not alluded to as having any connexion with the nerves of the uterus. In farther illustration of the history of this subject, Dr. Lee continues : " Dr. Baly states that he met with the following isolated passage in a paper by Schwann—the flat bands under the peritoneum were similar to those described by Lobstein. 'In the uterus of a woman who had borne a child at the full period of pregnancy, I found, immediately under the peritoneal coat, not muscular fibres with transverse striæ, but long very flat bands of different breadth, which presented very indistinct longitudinal striæ, (Langstreifung.) In some few, I could distinguish a flat, pale nucleus, very much elongated, and containing a smaller corpuscle.' "

We have seen from Dr. Lee's historical review that Dr. William Hunter believed that the nerves of the uterus enlarge during pregnancy, but that Mr. John Hunter distinctly denied this. Dr. Lee's dissections can scarcely leave any doubt that the nerves do enlarge during pregnancy, but as to how this enlargement takes place requires yet to be determined.

That the nerves of the uterus, after having performed their proper functions during gestation and labour, gradually return to the condition in which they are found in the unimpregnated uterus, Dr. Lee thinks is made certain by the following observation :

" On the 27th of June, 1840, I examined the uterus of a woman who died suddenly on the tenth day after delivery. The hypogastric plexus, and those both on the anterior and posterior surfaces of the body of the uterus, were very much reduced in size from what they were observed to be in the uteri of six and nine months." (p. 7.)

Dr. Lee concludes the account of his dissections of the human uterus with the following remarks :

" These dissections prove that the human unimpregnated uterus possesses a great system of nerves, which enlarges with the coats, blood-vessels, and absorbents during pregnancy, and which returns after parturition to its original condition before conception took place. It is chiefly by the influence of these nerves that the uterus performs the varied functions of menstruation, conception, and parturition, and it is solely by their means, that the whole fabric of the nervous system sympathises with the different morbid affections of the uterus. If these nerves of the uterus could not be demonstrated to exist, its physiology and pathology would be completely inexplicable." (p. 8.)

Nine dissections altogether of the nerves of the human uterus, both in the impregnated and the unimpregnated state, are recorded in the work before us; an account of a dissection of the nerves of the uterus in the mare is also given which will be found to present some interesting analogies.

---

